# Event-Related Desynchronization of MEG Alpha-Band Oscillations during Simultaneous Presentation of Audio and Visual Stimuli in Children with Autism Spectrum Disorder

**DOI:** 10.3390/brainsci13091313

**Published:** 2023-09-13

**Authors:** Vardan Arutiunian, Giorgio Arcara, Irina Buyanova, Olga Buivolova, Elizaveta Davydova, Darya Pereverzeva, Alexander Sorokin, Svetlana Tyushkevich, Uliana Mamokhina, Kamilla Danilina, Olga Dragoy

**Affiliations:** 1 Center for Child Health, Behavior and Development, Seattle Children’s Research Institute, 1920 Terry Ave., Seattle, WA 98101, USA; 2 IRCCS San Camillo Hospital, 70 Via Alberoni, Lido, 30126 Venice, Italy; giorgio.arcara@gmail.com; 3 Center for Language and Brain, HSE University, 3 Krivokolenny Pereulok, 101000 Moscow, Russia; irinamatulko@gmail.com (I.B.); bublixa@gmail.com (O.B.); odragoy@hse.ru (O.D.); 4 Federal Resource Center for ASD, Moscow State University of Psychology and Education, 19 Architectora Vlasova Str., 117335 Moscow, Russia; el-davydova@mail.ru (E.D.); dasha.pereverzeva@gmail.com (D.P.); sorokinab@mgppu.ru (A.S.); tyushkevichsv@yandex.ru (S.T.); uliana.mamokhina@gmail.com (U.M.); d-kk@mail.ru (K.D.); 5 Chair of Differential Psychology and Psychophysiology, Moscow State University of Psychology and Education, 2A Shelepikhinaskaya Naberezhnaya, 123290 Moscow, Russia; 6 Haskins Laboratories, 300 George St., New Haven, CT 06511, USA; 7 Scientific Research and Practical Center of Pediatric Psychoneurology, 74 Michurinskiy Prospekt, 119602 Moscow, Russia; 8 Institute of Linguistics, Russian Academy of Sciences, 1/1 Bolshoy Kislovsky Ln, 125009 Moscow, Russia

**Keywords:** autism spectrum disorder, magnetoencephalography, auditory cortex, event-related desynchronization (ERD), alpha power

## Abstract

Alpha-band (8–12 Hz) event-related desynchronization (ERD) or a decrease in alpha power in electro- and magnetoencephalography (EEG and MEG) reflects the involvement of a neural tissue in information processing. It is known that most children with autism spectrum disorder (ASD) have difficulties in information processing, and, thus, investigation of alpha oscillations is of particular interest in this population. Previous studies have demonstrated alterations in this neural activity in individuals with ASD; however, little is known about alpha ERD during simultaneous presentation of auditory and visual stimuli in children with and without ASD. As alpha oscillations are intimately related to attention, and attention deficit is one of the common co-occurring conditions of ASD, we predict that children with ASD can have altered alpha ERD in one of the sensory domains. In the present study, we used MEG to investigate alpha ERD in groups of 20 children with ASD and 20 age-matched typically developing controls. Simple amplitude-modulated tones were presented together with a fixation cross appearing on the screen. The results showed that children with ASD had a bilateral reduction in alpha-band ERD in the auditory but not visual cortex. Moreover, alterations in the auditory cortex were associated with a higher presence of autistic traits measured in behavioral assessment.

## 1. Introduction

Autism spectrum disorder (ASD) is a heterogeneous neurodevelopmental condition associated with a deficit in social interaction/communication and the presence of stereotype/repetitive behavior and restricted interests or an atypical response to sensor information [1]. Along with the core symptoms, children with ASD usually have co-occurring conditions, including medical problems [2], cognitive deficits [3], intellectual disability [4], impairments in attention [5], language [6], etc. Previous studies have shown alterations in multiple different functional neurobiological mechanisms in ASD, and some of them were related to both core and co-occurring conditions of this disorder [7,8,9]. In general, altered information processing in different brain areas/abnormal neural computations in response to external stimuli is considered one of the common neurobiological deficits in ASD individuals.

At the neural level, one of the possible neurophysiological mechanisms that can be related to difficulties in information processing is alterations in the cortical alpha-band oscillations (8–12 Hz) measured with electro- and/or magnetoencephalography, EEG and MEG [10,11]. According to the previous findings, alpha oscillations can be a non-invasive measure of the activation level in the brain due to their functional specificity [12,13,14]. In information processing, event-related desynchronization (ERD) at the alpha band is associated with the extent of the involvement of the cortical region in the task [15,16,17], so the higher suppression of alpha power (lower power) reflects more involvement of a neural tissue in the stimulus processing. On the other hand, event-related synchronization (ERS) at the same frequency band is related to filtering or ignoring external stimuli [18,19]. Importantly, the cortical mechanisms of both ERD and ERS are not area-specific, so the mechanisms of alpha power suppression/functional inhibition are the same in different sensory systems and modalities (auditory, visual, etc.). As alpha oscillations can regulate the extent of the involvement of a cortical region in stimulus processing, they are considered one of the neural markers of excitation (E)/inhibition (I) balance in the brain [20]. Thus, they are of particular interest in ASD research because E/I imbalance is now considered one of the possible pathophysiological mechanisms of ASD, according to animal models of autism [21,22], investigation of postmortem neural tissue of autistic brains [23,24] and cognitive experiments with ASD individuals [25,26].

Abnormalities in alpha-band oscillations were observed in children with ASD in different cortical regions during different tasks [27,28,29]. For example, in the study of Martínez et al. 2019 [30], using a motion processing task, altered alpha ERD was shown in the visual cortex in children with ASD when comparing them to controls; moreover, the amplitude of ERD correlated with the higher presence of autistic traits. In the auditory domain with a language task, the relationship between higher alpha-band ERD and better language skills of children with ASD was revealed, using a word/nonword comprehension task in MEG [31]. Finally, abnormal ERD of alpha oscillations has been demonstrated in different cortical areas of children with ASD in comparison to age- and non-verbal-IQ-matched typically developing (TD) controls, using an attentional capture task [32]. In general, one of the psychological functional explanations of the alteration in alpha power suppression during different tasks in ASD is the extent of the involvement of attention in the task. It is known that when attention is engaged in the stimulus processing, the power of alpha oscillations decreases, and there is a relationship between the extent of attention engagement and alpha power (i.e., more involvement of attention is associated with lower power) [33,34,35]. As impairment of attention is one of the common and widespread characteristics of the ASD population [5], probably, altered alpha ERD can be explained by less involvement of attention in the task.

Although there are a few studies addressing stimulus-induced alpha-band ERD in children with ASD, still little is known about alpha power suppression during simultaneous processing of stimulus from different modalities (i.e., auditory and visual). Previous studies have revealed atypical event-related and evoked potentials in ASD individuals when giving them high-order attention and language tasks which require parallel cortical processing and simultaneous activation in different sensory modalities [36,37]. However, the extent of alpha-band ERD in response to basic stimuli of different modalities presented simultaneously in a completely passive paradigm is still largely unexplored in the ASD population. At the same time, this approach has a great advantage in comparison to task-related approaches because it allows us to include in neuroimaging research children with profound autism who have severe intellectual disability and who are minimally or fully non-verbal. Moreover, it is not very well understood if these abnormalities in basic processing (if any) would be related to the clinical characteristics of children with ASD. We aim to fill this gap.

The goal of the present study is to investigate MEG alpha-band ERD in the primary/secondary auditory and visual cortices in the group of primary-school-aged children with ASD. We use a passive paradigm that does not require any response from children, presenting them with simple auditory stimuli (amplitude-modulated tones) and visual stimuli (fixation cross on the screen) simultaneously. As alpha-band neural oscillations are associated with attention, and attention deficit is one of the common co-occurring conditions of ASD, we predict that children with ASD can have altered alpha ERD in one of the sensory domains. Additionally, we address the relationship between alpha ERD and clinical characteristics of the ASD group.

## 2. Materials and Methods

### 2.1. Recruitment

A total of 40 children participated in the present study: 20 children with ASD (5 girls, age range 8.02–14.01 years, *M*_age_ = 10.03, *SD* = 1.7) and 20 TD children as a control group (9 girls, age range 7.02–12.03 years, *M*_age_ = 9.11, *SD* = 1.3). All participants had normal hearing and normal or corrected-to-normal vision. This is the same group of children who took part in the previous study [7].

*ASD diagnosis.* The diagnosis of children with ASD was based on the criteria of the International Classification of Diseases–10 [38], and 18 out of 20 children were assessed with Autism Diagnosis Observation Schedule–Second Edition, ADOS-2 [39]. Additionally, parents of both groups of children completed the Russian version of the Autism Spectrum Quotient: Children’s Version, AQ [40]. AQ is a questionnaire for assessing social and communication abilities of a child which allows one to obtain standard scores in five different “scales” referring to autism and broader phenotype (social abilities, communication, attention to detail, attention switching and imagination). The validity of the diagnosis was confirmed also by total AQ score where higher score is related to higher presence of autistic traits.

*Language abilities.* Language skills of both groups of children were assessed with the Russian Child Language Assessment Battery [41]; as a result, three standard scores were calculated for each child, i.e., mean language score (MLS), language production score (LPS) and language comprehension score (LCS).

*Non-verbal intelligence.* The non-verbal IQ of children with ASD was measured with the Kaufman Assessment Battery for Children, NVI index [42] or the Wechsler Intelligence Scale for Children–Third Edition, performance IQ score [43]; the non-verbal intelligence of TD children was assessed with the Raven’s Colored Progressive Matrices [44].

See Table 1 for demographic information and group comparisons.

### 2.2. Ethical Approval

The study was approved by the ethics committee of Moscow State University of Psychology and Education (for ASD group) and the HSE University Committee on Interuniversity Surveys and Ethical Assessment of Empirical Research (for TD group) and was conducted according to the Declaration of Helsinki. Parents of all children signed a written consent form.

### 2.3. Stimuli and Procedure

**Figure 1:**
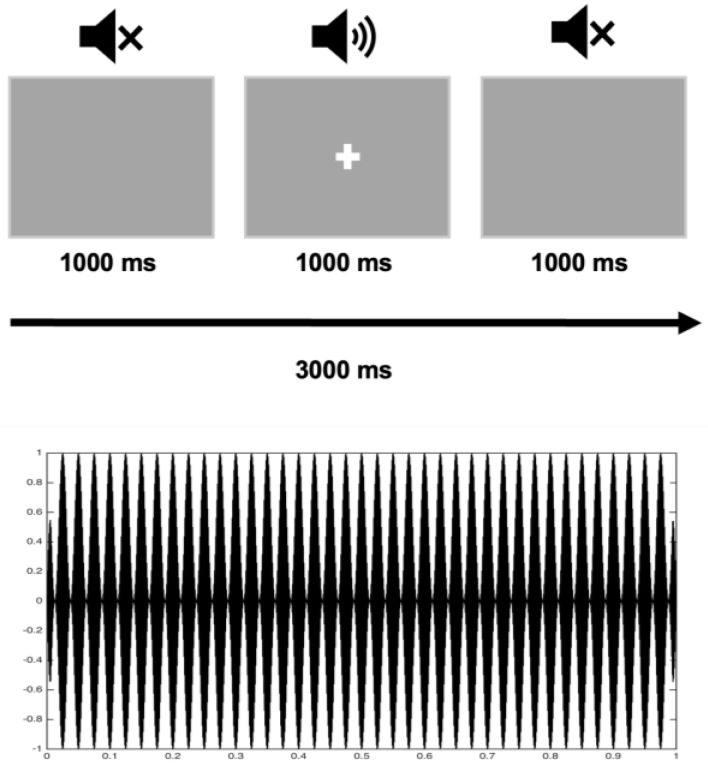
The structure of the experiment: a stimulus presentation (**upper** panel) and amplitude-modulated tone (**lower** panel).

The audio stimuli were simple amplitude-modulated tones with 1000 ms duration (carrier frequency set to 1000, sample rate of 44,100 Hz and amplitude modulation at 40 Hz) and a visual fixation cross. The presentation of audio stimuli (n = 90) was binaural with 2000 ms inter-trial interval, and they were presented during one ~5 min block. We used PsychoPy software version 1.90.2 [45] for stimuli presentation. Audio stimuli were delivered via plastic ear tubes with foam tips inserted into the ear canals, and the intensity level was set at 83.7 dB sound pressure level. Fixation cross appeared on the screen at the same time as the presentation of audio stimuli (Figure 1).

### 2.4. Structural Magnetic Resonance Imaging (MRI) Acquisition

In order to build individual head models and provide source estimation, we collected structural MRI data with a 1.5 T Siemens Avanto scanner with the following parameters: repetition time = 1900 ms, echo time = 3.37 ms, flip angle = 15°, matrix size = 256 × 256 × 176, and voxel size = 1.0 × 1.0 × 1.0 mm^3^. We used the FreeSurfer software version 7.1.0 [46] for MRI segmentation and reconstruction of cortical surface (it was down-sampled to 15,000 vertices for each participant). The co-registration between MRI and MEG was performed with the Brainstorm toolbox [47] based on the six reference points (left and right pre-auricular points, nasion, anterior and posterior commissure and interhemispheric point) and the additional digitized head points (*N* = ~150).

We were able to collect MRI for all TD children and 15 out of 20 children with ASD. For children with ASD who did not tolerate scanning procedure, we used the template anatomy (“MRI: ICBM152”) applying the special warping procedure implemented in Brainstorm. This algorithm allowed us to build a pseudo-individual brain based on the head points digitized before the MEG data collection and represent the real head shape of each child.

### 2.5. MEG Data Collection and Pre-Processing

MEG data were acquired in a sitting position in a magnetically shielded room with a whole-head 306-channel MEG system (Vectorview, Elekta Neuromag) which consists of 204 orthogonal planar gradiometers and 102 magnetometers. The head position in the MEG helmet was monitored every 4 ms during the recording via four head position indicator (HPI) coils which were digitized together with fiducial points with the 3D digitizer “Fastrak” (Polhemus). The *temporal signal space separation* [48] and the movement compensation approaches implemented in MaxFilter were applied. We used an electrooculogram (EOG) to detect the blinks (the electrodes were placed above and below the left eye) and horizontal eye movements (the electrodes were placed at the left and right outer canthi). An electrocardiography (ECG) was monitored with ECG electrodes to compensate for cardiac artifacts.

MEG was collected at 1000 Hz sampling rate, and the offline band-pass filter of 0.1–330 Hz and the notch filter of 50 Hz were applied. We used only gradiometers for the analysis because of the current debates over mixing both magnetometers and gradiometers, which have different levels of noise (see [49]). Ocular and cardiac artifact removal was performed with EEGLAB’s [50] independent component analysis (ICA) implemented in Brainstorm. Cleaned MEG data were segmented in 3000 ms epochs with 1500 ms before and after stimulus onset. The DC offset correction of from −100 ms to −2 ms was applied. Additionally, epochs were inspected visually, and those affected by muscular artifacts were rejected. The number of artifact-free epochs did not differ between groups of children: TD group, *M*_number_ = 84, *SD* = 3.30, range 77–87; ASD group, *M*_number_ = 84, *SD* = 3.85, range 75–87; *t*(37.13) = 0.17, *p* = 0.86.

### 2.6. MEG Source Localization

In order to compute individual head models, we used the “Overlapping spheres” method [51]. The inverse problem was solved with the depth-weighted linear L2-minimum norm estimate method [52], with the dipole orientation constrained to be normal to the cortical surface. A common imaging kernel was computed and then applied to obtain single epoch cortical reconstructions. A noise covariance was estimated from a 2 min empty room recording, taken after each participant’s recording session. To provide a between-subject comparison, the individual MNEs were projected to the “MRI: ICBM152” template brain.

The regions of interest (ROIs) were primary/secondary auditory and visual areas in both hemispheres corresponding to 41 and 42 Broadman’s areas (for the auditory cortex) and 17, 18 and 19 Broadman’s areas (for the visual cortex). We performed time-frequency (TF) analysis at the source level using Morlet wavelets (central frequency = 10 Hz, time resolution = 0.5 s) to calculate the power. TF maps were normalized with an event-related synchronization/desynchronization approach considering the time window between −500 ms and −200 ms as a baseline, evaluating the deviation from the mean over the baseline, based on the following formula: (x − mean)/mean × 100. Such a time window was chosen to avoid the edge effects. The normalized power was averaged in the standard alpha-band frequency range (8–12 Hz) in the interval from 200 ms to 1000 ms after stimulus onset. To establish the cortical sources of alpha desynchronization in the auditory and visual cortices, we estimated MNI coordinates of 60 vertices with the highest alpha power decrease values in the defined ROIs (15 vertices per ROI) and, for further analysis, extracted power averaged over these 15 vertices in the time interval between 200 ms and 1000 ms in each hemisphere for each child. This approach was successfully used in the previous studies because it accounted for inter-individual variability of neural responses which contributes to a more precise source localization [7,53].

### 2.7. Statistical Analysis

Statistical analysis was performed in *RStudio* [54], using linear models with and without mixed effects with *lme4* [55]. For data visualization, we used *ggplot2* package [56]. The structures of the models will be specified further.

## 3. Results

### 3.1. Phenotypical Characteristics of Participants

Although children with and without ASD did not differ in age, there was a difference in language scores as well as the presence of autistic traits (see Table 1). The group of children with ASD was heterogeneous and consisted of both low- and high-functioning children.

### 3.2. Localization of Neural Responses

**Figure 2:**
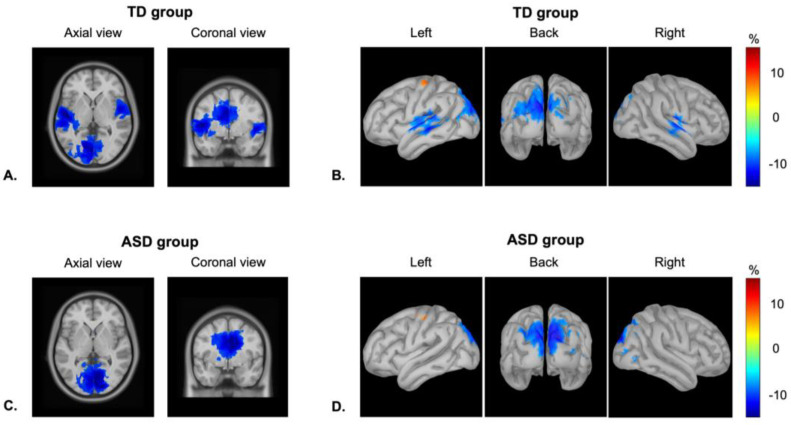
Alpha-band even-related desynchronization, power, % change from the baseline: axial and coronal views of alpha power decrease in (**A**) TD children and (**C**) children with ASD; cortical distribution of activity at the 8–12 Hz frequency range in (**B**) TD children and (**D**) children with ASD (the amplitude threshold is set to 70% of highest values).

Figure 2 represents the patterns of alpha-band ERD in the temporal and visual cortices in both groups of children averaged between 200 and 1000 ms from the stimuli onsets. As was expected, the highest values of power decrease at the alpha range were observed in the primary/secondary temporal and occipital regions in both hemispheres. The visual exploration of neural responses demonstrated a clear alpha-band ERD in the occipital regions in both groups of children. By contrast, in the temporal cortex, there was clear alpha power suppression in the TD group in both hemispheres, whereas in the ASD group, there was no prominent activity in these regions.

### 3.3. Between-Group Comparisons of Neural Responses

In order to compare alpha-band ERD between groups of children in the auditory and visual cortices, we fitted two linear mixed-effects models (the first model with the auditory alpha power and the second one with the visual alpha power) with neural activity as a dependent variable, main effects of group (ASD vs. TD), hemisphere (left vs. right) and group × hemisphere interaction as fixed effects and participants as a random intercept, based on the following structure: *lmer(neural activity ~ 1 + group + hemisphere + group* × *hemisphere + (1 | ID), data = data, control = lmerControl(optimizer = “bobyqa”))*.

The results of the first model showed a statistically significant main effect of group (alpha power was higher in the ASD group) but no effects of hemisphere or group × hemisphere interaction (Table 2).

The second model did not reveal any statistically significant effects (Table 3).

**Figure 3:**
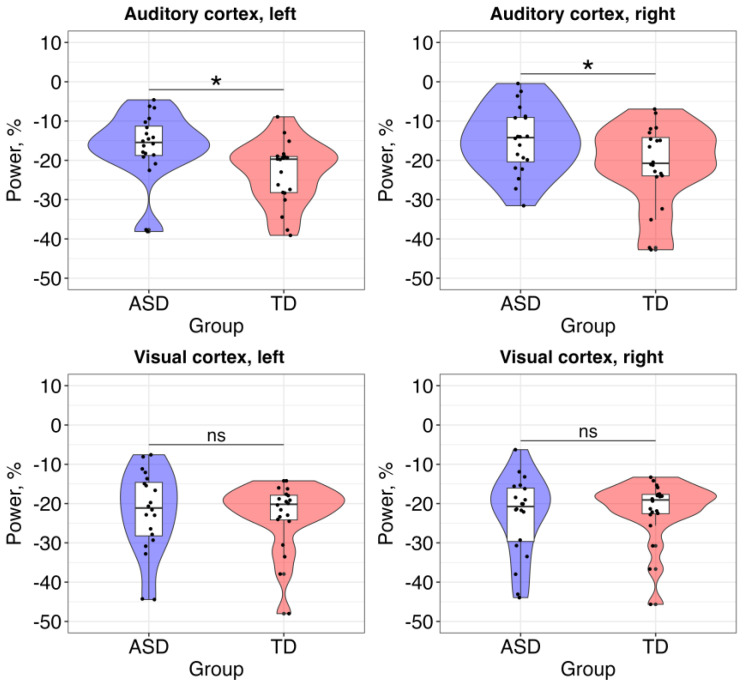
Between-group comparisons of the alpha-band neural activity in the auditory and visual cortices. Significance is labeled with *; ns = non-significant.

To summarize, atypical neural response at the alpha band was observed only in the auditory cortex of children with ASD (Figure 3).

### 3.4. The Relationships between Alpha Activity in the Auditory and Visual Cortices

Previous studies have shown that the alpha-band ERD depends on the involvement of attention in the perception/processing of a stimulus [19], and simultaneous presentation of auditory and visual stimuli can influence alpha power. To address this, we focused on two questions: (1) how alpha-band ERD in the auditory cortex was related to alpha-band ERD in the visual cortex, and (2) if there was a difference between alpha ERDs in auditory and visual areas. For that, we fitted two linear models for both hemispheres. The first model included visual alpha ERD as a dependent variable, auditory alpha ERD as a predictor and the group as a nested effect to account for possible different developmental trajectories (see [57]), according to the following structure: *lm(visual alpha ~ 1 + group/auditory alpha, data = data)*. The second model consisted of alpha ERD as a dependent variable, region of interest (auditory vs. visual) as the main effect, the group as a nested effect and participants as a random intercept: *lmer(power ~ 1 + group/region + (1 | ID), data = data, control = lmerControl(optimizer = “bobyqa”))*.

The results of both models revealed similar patterns. In the TD group of children, there was no relationship between auditory and visual alpha ERD in both hemispheres: *left hemisphere*, β = 0.32, SE = 0.25, *t* = 1.92, *p* = 0.20; *right hemisphere*, β = −0.15, SE = 0.18, *t* = −0.81, *p* = 0.42. By contrast, in children with ASD, there was a positive relationship between neural responses in both hemispheres, so higher power in the auditory cortex was associated with higher power in the visual cortex: *left hemisphere*, β = 0.70, SE = 0.22, *t* = 3.11, *p* = 0.003; *right hemisphere*, β = 0.70, SE = 0.22, *t* = 3.11, *p* = 0.003. Moreover, the results showed no difference in the TD group between auditory and visual areas in alpha ERD: *left hemisphere*, β = −0.24, SE = 2.10, *t* = −0.11, *p* = 0.91; *right hemisphere*, β = 0.69, SE = 2.63, *t* = 0.26, *p* = 0.79. However, in the ASD group, alpha ERD was higher in the visual cortex in comparison to the auditory area in both hemispheres: *left hemisphere*, β = 5.63, SE = 2.10, *t* = 2.67, *p* = 0.01; *right hemisphere*, β = 8.18, SE = 2.63, *t* = 3.11, *p* = 0.003. These findings hypothetically can suggest that the attention of children with ASD was more concentrated on visual stimuli which causes less effective processing of auditory stimuli.

### 3.5. The Relationships between Alpha ERD and Symptom Severity in Children with ASD

In order to investigate how alpha-band ERD in the auditory and visual areas is related to the severity of autistic traits in children with ASD, we fitted two linear mixed-effects models with the neural activity as a dependent variable (auditory/visual ERD), the main effects of AQ “scales” associated with attention and core symptoms of autism (social skills, communication, attention to detail and attention switching), hemisphere as a nested effect and participants as a random intercept as follows: *lmer (neural activity~1 + hemisphere/AQ social abilities + hemisphere/AQ communication + hemisphere/AQ attention to detail + hemisphere/AQ attention switching + (1|ID), data = data, control = lmerControl (optimizer = “bobyqa”))*.

**Figure 4:**
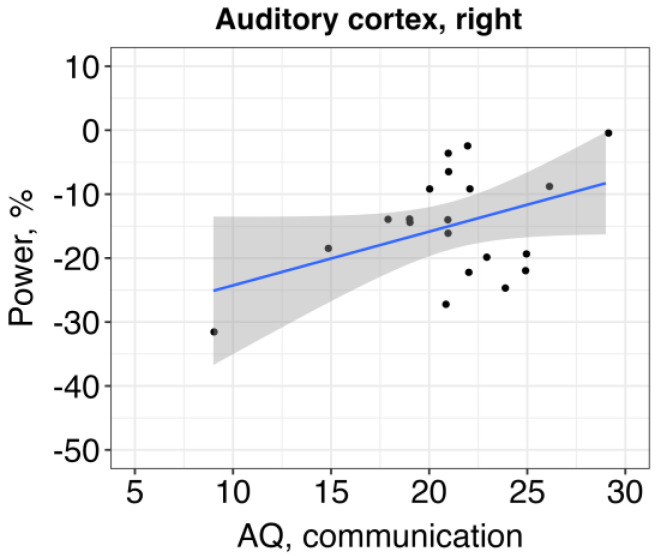
The relationship between auditory alpha ERD in the right hemisphere and communication skills of children with ASD (higher AQ score refers to a higher presence of autistic traits).

The results revealed a relationship between alpha ERD in the right auditory cortex and communication skills, so that the higher alpha power (and, subsequently, more altered ERD) was associated with more impaired communication: β = 1.54, SE = 0.74, *t* = 2.09, *p* = 0.04 (Figure 4). Other effects were non-significant (Table 4 and Table 5).

## 4. Discussion

In general, the current study addressed MEG alpha-band ERD in the auditory and visual cortices of primary-school-aged children with ASD. We used a passive perception paradigm with the simultaneous presentation of simple auditory and visual stimuli, i.e., amplitude-modulated tones and a fixation cross. Additionally, we focused on the relationship between alpha-band ERD and clinical characteristics of children with ASD. The study revealed altered power suppression at the alpha-band neural oscillations in the auditory but not visual cortex in children with ASD, and this abnormal suppression in the right hemisphere was related to more severe autistic traits.

The analysis of source localization in MEG showed that the cortical generators of alpha-band ERD were in the primary/secondary auditory and visual cortices in the left and right hemispheres. This corresponded to the previous studies, which revealed that the sources of sustained auditory response are located in A1/A2 [7,53], and the sources of a simple visual response are in V1/V2 [58]. The comparison of alpha-band ERD in the auditory and visual cortices in children with and without ASD revealed abnormal bilateral activity in the ASD group in the auditory but not visual cortex, indicating that the suppression of alpha power was higher in the TD group. These results are in line with the previous findings that showed altered alpha power suppression during information processing in different cortical areas in ASD [27,28,29,30,31,32]. Lower suppression of alpha power in the auditory cortex of children with ASD can be hypothetically related to E/I imbalance [21]: studies with both animals and humans have shown that the extent of alpha suppression during stimulus processing is associated with the extent of the involvement of cortical area in the activity [19]. Therefore, abnormal alpha-band ERD in the auditory cortex of ASD individuals can be related to less involvement of the cortical area in the processing.

Neural activity at the alpha band during visual processing in comparison to auditory processing did not differ between groups of children. Hypothetically, it can be explained by the fact that children with ASD were more involved in the visual but not auditory task. Previous studies have demonstrated atypical brain activation in ASD individuals when giving them tasks that require simultaneous activation in different sensory modalities [36,37]. Indeed, in children with ASD (but not in TD children), we observed an atypical relationship between alpha power in the auditory and visual areas: the higher power in the auditory area was related to higher power in the visual area. Yet, alpha ERD was higher in the visual cortex of children with ASD in comparison to the auditory cortex. Given that the extent of alpha power suppression is associated with the extent of the involvement of attention in the task [33,34,35], we hypothesize that the engagement of the visual cortex in information processing influenced the efficient processing in the auditory cortex.

Atypical alpha-band ERD in the auditory cortex of children with ASD had a clinical relevance/consequence. In the right hemisphere, more altered alpha power (i.e., higher power) was related to the higher presence of autistic traits on the “communication” scale, so that ASD individuals with less efficient power suppression in the right temporal region had worse communication skills measured with AQ. This finding is supported by previous studies that showed the involvement of the right hemisphere in language and communication in autism [59,60]. The novelty of our finding is that the low-level neural activity in the right hemisphere is also associated with the high-order cognitive and communication abilities of children with ASD.

The study has some limitations that should be mentioned here. First, we hypothesized the involvement of attention in the auditory and visual tasks but did not test it directly due to the structure of the experiment. In order to answer that question directly, it is needed to conduct an additional experiment that will consist of three different blocks: the first one is only with auditory stimuli, the second one is only with visual stimuli, and the third one is with both types as in the current study. If in the first and second blocks, alpha ERD does not differ between groups of children, and only in the third block, there is a between-group difference in one of the domains, which means that attention plays a role in the simultaneous processing of auditory and visual stimuli in children with ASD. Second, ASD and TD groups of children had different male/female ratios (25% and 45% of girls in ASD and TD groups, respectively). As biological sex can influence neural activity in ASD (see [61]), it would be beneficial to include an equal number of boys and girls in future studies.

## 5. Significance

Our environment is dynamic, and normal information perception and processing require the simultaneous involvement of different sensory modalities [36,37,62]. This, subsequently, needs normal functioning of different brain areas. However, simultaneous processing of information from different modalities can be altered in children with ASD due to their attention limitations [5]. In this study, we showed that the perception of even simple auditory and visual stimuli presented at the same time can be impaired in children with ASD. This finding can have not only clinical but also pedagogical relevance, pointing to the way therapists/teachers need to work with children with ASD. If autistic individuals cannot pay attention at the same time to stimuli from different sensory modalities, therapists/teachers need to interact with these children considering this specific-to-ASD way of information processing. Taking into account this way of functioning of the autistic brain and organizing the learning process in a proper-to-them way will contribute to better outcomes for these children.

## 6. Conclusions

Children with ASD had an atypical alpha-band ERD in the auditory but not visual cortex in response to basic auditory and visual stimuli presented simultaneously. This, hypothetically, can be related to more involvement of attention to the visual task which influenced the efficient processing in the auditory cortex. Moreover, atypical suppression of alpha power in the right auditory area of children with ASD was associated with the higher presence of autistic traits on the “communication” scale. Our study revealed some low-level alterations in individuals with ASD and the relevance of the identified alterations to the clinical characteristics of this population.

## Figures and Tables

**Table 1:** Demographic information of participants, M ± SD.

Characteristics	ASD	TD	*t*	*p*
Age (years)	10.03 ± 1.7	09.11 ± 1.3	0.70	0.48
AQ total	83.6 ± 18.8	50.2 ± 14.2	6.23	<0.001 ***
AQ social abilities	15.9 ± 6.0	7.6 ± 3.0	5.50	<0.001 ***
AQ communication	21.1 ± 4.2	8.6 ± 4.7	8.94	<0.001 ***
AQ attention to detail	14.9 ± 4.9	12.8 ± 4.9	1.37	0.17
AQ attention switching	16.2 ± 4.0	12.3 ± 3.0	3.39	0.001 **
AQ imagination	15.4 ± 6.4	8.9 ± 3.1	4.07	<0.001 ***
MLS	0.75 ± 0.23	0.95 ± 0.02	−4.04	<0.001 ***
LPS	0.76 ± 0.24	0.96 ± 0.02	−3.66	0.001 **
LCS	0.73 ± 0.24	0.95 ± 0.03	−4.07	<0.001 ***
Non-verbal IQ	85.4 ± 17.9	31.8 ± 2.7	–	–

*Note: T*-tests were provided to compare the characteristics of ASD and TD groups of children. The significance is labeled with * *p* < 0.05, ** *p* < 0.01 and *** *p* < 0.001 and highlighted in bold.

**Table 2:** Between-group comparisons in alpha power in the auditory cortex.

Predictor	Estimate	Standard Error	*t*	*p*
(Intercept)	−18.95	1.29	−14.71	<0.001 ***
Group	3.23	1.29	2.51	0.01 *
Hemisphere	−0.95	0.57	−1.66	0.10
Group × hemisphere	0.13	0.57	0.22	0.83

*Note:* The significance is labeled with * *p* < 0.05, ** *p* < 0.01 and *** *p* < 0.001 and highlighted in bold.

**Table 3:** Between-group comparisons in alpha power in the visual cortex.

Predictor	Estimate	Standard Error	*t*	*p*
(Intercept)	−22.52	1.42	−15.87	<0.001 ***
Group	−0.11	1.42	−0.07	0.94
Hemisphere	−0.08	0.43	−0.19	0.85
Group × hemisphere	0.53	0.43	1.24	0.22

*Note:* The significance is labeled with * *p* < 0.05, ** *p* < 0.01 and *** *p* < 0.001 and highlighted in bold.

**Table 4:** The relationship between alpha ERD in the auditory cortex and clinical characteristics of children with ASD.

**Predictor**	**Estimate**	**Standard Error**	***t***	***p***
(Intercept)	−31.03	11.02	−2.82	0.009 **
Hemisphere	0.58	10.03	0.05	0.95
Hemisphere (left)/AQ social abilities	−0.32	0.47	−0.69	0.49
Hemisphere (right)/AQ social abilities	−0.38	0.47	−0.81	0.43
Hemisphere (left)/AQ communication	1.28	0.74	1.73	0.10
Hemisphere (right)/AQ communication	1.54	0.74	2.09	0.04 *
Hemisphere (left)/AQ attention to detail	0.07	0.52	0.14	0.89
Hemisphere (right)/AQ attention to detail	−0.19	0.52	−0.36	0.79
Hemisphere (left)/AQ attention switching	−0.52	0.71	−0.74	0.47
Hemisphere (right)/AQ attention switching	−0.51	0.71	−0.72	0.48

*Note:* The significance is labeled with * *p* < 0.05, ** *p* < 0.01 and *** *p* < 0.001 and highlighted in bold.

**Table 5:** The relationship between alpha ERD in the visual cortex and clinical characteristics of children with ASD.

**Predictor**	**Estimate**	**Standard Error**	***t***	***p***
(Intercept)	−35.36	14.10	−2.51	0.02 *
Hemisphere	3.01	7.75	0.39	0.70
Hemisphere (left)/AQ social abilities	−0.32	0.60	−0.54	0.60
Hemisphere (right)/AQ social abilities	−0.50	0.60	−0.83	0.42
Hemisphere (left)/AQ communication	0.36	0.95	0.39	0.70
Hemisphere (right)/AQ communication	0.80	0.95	0.85	0.41
Hemisphere (left)/AQ attention to detail	0.48	0.67	0.71	0.48
Hemisphere (right)/AQ attention to detail	0.79	0.67	1.17	0.26
Hemisphere (left)/AQ attention switching	0.21	0.91	0.23	0.81
Hemisphere (right)/AQ attention switching	0.70	0.91	−0.78	0.45

*Note:* The significance is labeled with * *p* < 0.05, ** *p* < 0.01 and *** *p* < 0.001 and highlighted in bold.

## Data Availability

The datasets generated and analyzed during the current study are not publicly available as they are human data but are available from the corresponding author on reasonable request.
